# βα-Hairpin Clamps Brace βαβ Modules and Can Make Substantive Contributions to the Stability of TIM Barrel Proteins

**DOI:** 10.1371/journal.pone.0007179

**Published:** 2009-09-29

**Authors:** Xiaoyan Yang, Sagar V. Kathuria, Ramakrishna Vadrevu, C. Robert Matthews

**Affiliations:** Department of Biochemistry and Molecular Pharmacology, University of Massachusetts Medical School, Worcester, Massachusetts; Griffith University, Australia

## Abstract

Non-local hydrogen bonding interactions between main chain amide hydrogen atoms and polar side chain acceptors that bracket consecutive βα or αβ elements of secondary structure in αTS from *E. coli*, a TIM barrel protein, have previously been found to contribute 4–6 kcal mol^−1^ to the stability of the native conformation. Experimental analysis of similar βα-hairpin clamps in a homologous pair of TIM barrel proteins of low sequence identity, IGPS from *S. solfataricus* and *E. coli*, reveals that this dramatic enhancement of stability is not unique to αTS. A survey of 71 TIM barrel proteins demonstrates a 4-fold symmetry for the placement of βα-hairpin clamps, bracing the fundamental βαβ building block and defining its register in the (βα)_8_ motif. The preferred sequences and locations of βα-hairpin clamps will enhance structure prediction algorithms and provide a strategy for engineering stability in TIM barrel proteins.

## Introduction

The (βα)_8_, TIM barrel is one of the most common folds in biology, supporting a myriad of catalytic functions essential to life [1]. Experimental [2] and bioinformatics [1], [3], [4] analyses of TIM barrel proteins have led to the conclusion that a pair of adjacent parallel β-strands and the intervening anti-parallel α-helix, i.e., the βαβ module, serve as the minimal unit of stability. Gene duplication of this elemental βαβ building block into higher-order structures has been suggested to result in several common βα-repeat structures, including the TIM barrel, Rossman, flavodoxin and leucine-rich folds [3]. The interactions stabilizing this super-secondary structure include: (1) main chain-main chain (MC–MC) hydrogen bonds (H-bonds) between the β-strands, (2) intra-helical MC–MC H-bonds, (3) hydrophobic interactions between the side chains (SC) protruding from the β-strands and the α-helix, (4) side chain-side chain (SC–SC) H-bonds and salt bridges, (5) dipole-dipole interactions between the α-helix and the pair of β-strands on which it docks [5] and (6) main chain-side chain (MC–SC) H-bonds [6]–[8]. The surprising role of a subset of non-local MC–SC H-bond interactions in structure and stability is the subject of this communication.

A majority of MC–SC interactions in proteins are local in sequence, usually within six residues [7], [8], and are often involved in capping either the N- or the C-termini of α-helices [9], [10]. Mutational analysis has shown that these non-covalent interactions usually contribute modestly, typically in the range of 1–2 kcal mol^−1^, to the stability of their resident proteins [11]–[14]. In contrast, the removal of three non-local MC–SC H-bond interactions each reduce the stability of the alpha subunit of tryptophan synthase (αTS), a TIM barrel protein, by 4–6 kcal mol^−1^, and disrupt the complete formation of the TIM barrel motif [6]. These three interactions in αTS, between MC amide H-bond donors and SC H-bond acceptors, connect the N-terminus of one element of secondary structure, either β-strand or α-helix, to the C-terminus of the subsequent element of structure, either α-helix or β-strand, respectively. These non-local MC-SC interactions were designated as βα-hairpin clamps and αβ-hairpin clamps, respectively [6]. The significant contribution to structure and stability by three such clamps in αTS [6] raises the possibility that potent βα- and αβ-hairpin clamps may be an important general feature of TIM barrel proteins.

A two-pronged approach was taken to probe the significance of βα-hairpin clamps in TIM barrel proteins. First, mutational analysis of two representative TIM barrel proteins, indole-3-glycerol phosphate synthase (IGPS) from *S. solfataricus* (sIGPS) and *E. coli* (eIGPS), shows that a subset of their βα-hairpin clamps make significant contributions to protein stability. Second, a survey of 71 TIM barrel proteins [15] explored the frequency, location and sequence preferences of all βα-hairpin clamps. The observed preferences for location and sequence for the βα-hairpin clamps and their contribution to the structure and stability of TIM barrel proteins suggest that the recognition of these interactions can enhance protein structure prediction algorithms and provide a strategy for engineering stability in TIM barrel proteins.

## Results

### Experimental analysis of βα-hairpin clamp interactions in two TIM barrel proteins

**Figure 1:**
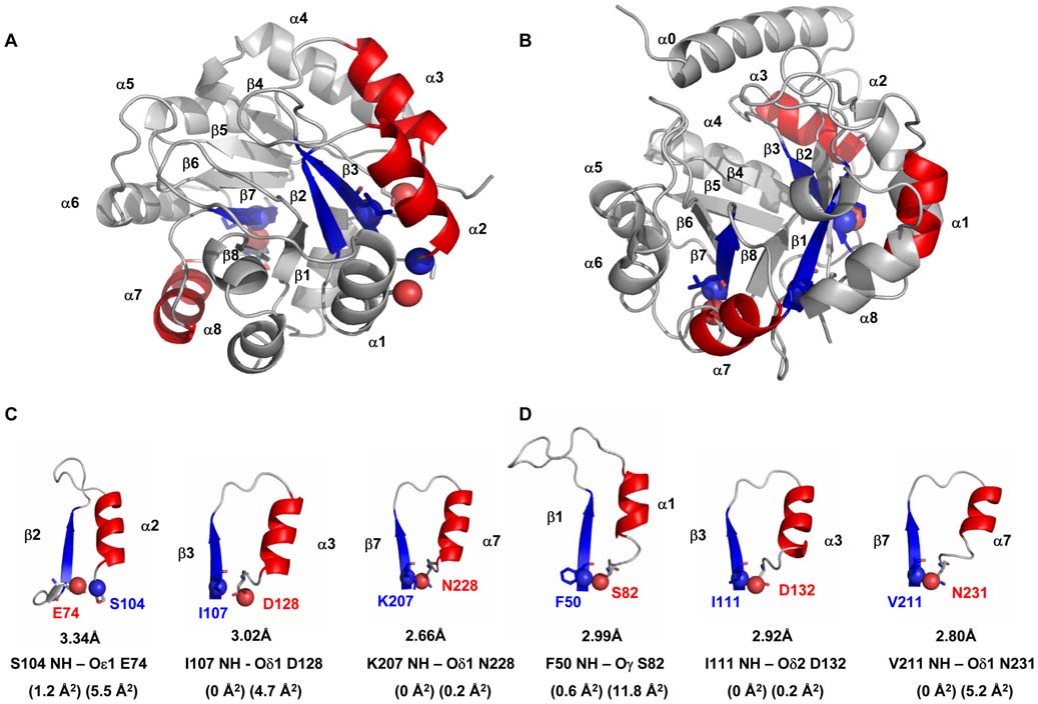
Ribbon diagrams of sIGPS (A) and eIGPS (B) highlighting the βα-hairpin clamps. (C) and (D) display the intervening elements of secondary structures between the residues forming the clamps for sIGPS: sIGPS-β2α2-S104_NH_→_Oε1_E74; sIGPS-β3α3-I107_NH_→_Oδ1_D128; and sIGPS-β7α7-K207_NH_→_Oδ2_N228 and for eIGPS: eIGPS-β1α1-F50_NH_→_Oγ_S82; eIGPS-β3α3-I111_NH_→_Oδ2_D132; and eIGPS-β7α7-V211_NH_→_Oδ1_N231. The SCs involved in the clamp interactions are highlighted with the H-bond donor and acceptor atoms shown in blue and red, respectively. The distances between the donor and acceptor atoms are indicated. The solvent exposed surface areas of the H-bond donor and acceptor atoms is shown in parenthesis. The H-bonds and their corresponding distances were determined by using the program HBPLUS [45]. The structures were generated using PyMOL *v* 0.99 [46], and the PDB codes are 2C3Z for sIGPS [21] and 1PII for eIGPS [22].

The generality of the potent hairpin clamps found in αTS was tested by mutational analysis of βα-hairpin clamps in two homologous TIM barrel proteins with low sequence identity (<30%) to αTS and to each other. sIGPS (Figure 1A) and eIGPS (Figure 1B), each contain three βα-hairpin clamps (Figure 1C and 1D), some of which are conserved in location with those in αTS and others between sIGPS and eIGPS. Figure 1 displays the distances between the donor and acceptor atoms of the βα-hairpin clamps interactions observed in sIGPS (Figure 1C) and eIGPS (Figure 1D). The solvent-exposed surface area of the H-bond acceptor atoms ranges from 0.2 Å^2^ (0%) to 11.8 Å^2^ (∼25%), while the MC H-bond donor amide is typically completely excluded from solvent (Figure 1C and 1D). The β1α1 clamp is observed in αTS and eIGPS (αTS-β1α1-F19_NH_→_Oδ2_D46, eIGPS-β1α1-F50_NH_→_Oγ_S82), the β2α2 clamp only appears in sIGPS (sIGPS-β2α2-S104_NH_→_Oε1_E74), the β3α3 clamp is observed in all three proteins (αTS-β3α3-I97_NH_→_Oδ2_D124, sIGPS-β3α3-I107_NH_→_Oδ1_D128 and eIGPS-β3α3-I111_NH_→_Oδ2_D132), and the β7α7 clamp is observed in sIGPS and eIGPS (sIGPS-β7α7-K207_NH_→_Oδ2_N228 and eIGPS-β7α7-V211_NH_→_Oδ1_N231).

#### Perturbation of the secondary and tertiary structure by clamp deletion in sIGPS and eIGPS

**Figure 2:**
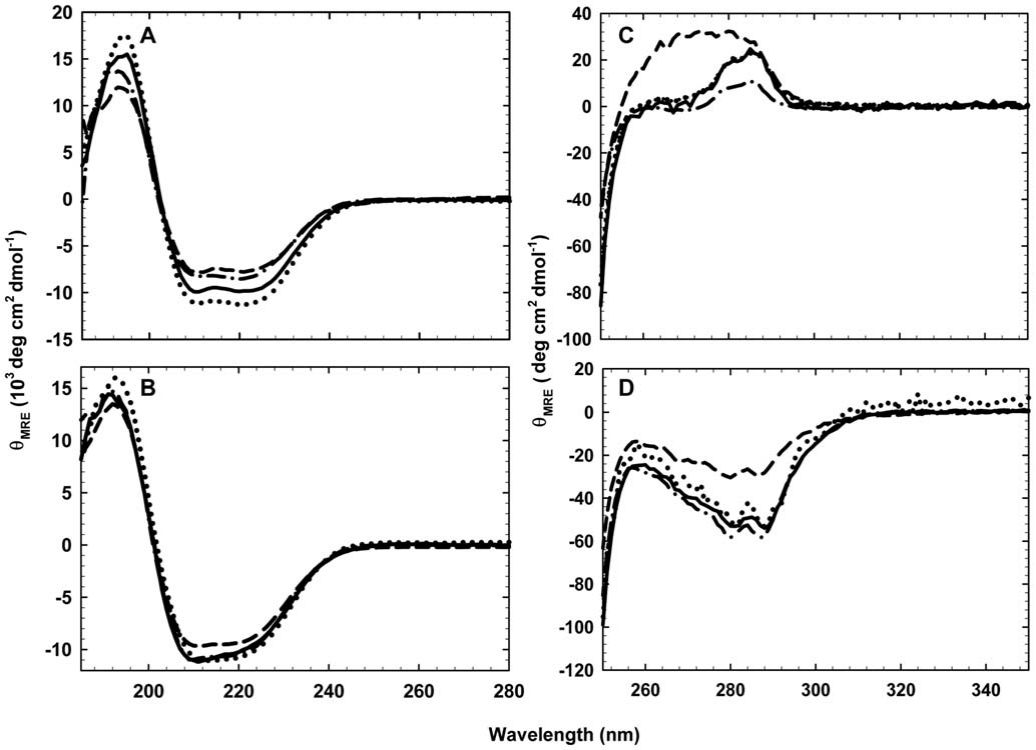
Ellipticity of wild-type and clamp-deletion variants of sIGPS and eIGPS. Far-UV (a, b) and near-UV (c, d) CD spectra of sIGPS (a) and (c): sIGPS-WT (–––––), sIGPS-Δβ2α2-E74A (– • –), sIGPS-Δβ3α3-D128A (•••), and sIGPS-Δβ7α7-N228A (– –); and eIGPS (b) and (d): eIGPS-WT (–––––), eIGPS-Δβ1α1-S82A (– • –), eIGPS-Δβ3α3-D132A (•••), and eIGPS-Δβ7α7-N231A (– –). Buffer conditions: 10 mM potassium phosphate, 0.2 mM K_2_EDTA, 1 mM βME, pH 7.8 for sIPGS and pH 7.0 for eIGPS at 25°C.

The contribution of each βα-clamp interaction to the structure of the TIM barrel proteins, sIGPS and eIGPS, was assessed by replacing the H-bond acceptor SC with alanine and monitoring the effects on the secondary and tertiary structure by far-UV and near-UV circular dichroism (CD) spectroscopy. The far-UV CD spectra for the wild-type (WT) and clamp-deletion variants of sIGPS (sIGPS-WT, sIGPS-Δβ2α2-E74A, sIGPS-Δβ3α3-D128A and sIGPS-Δβ7α7-N228A) and eIGPS (eIGPS-WT, eIGPS-Δβ1α1-S82A, eIGPS-Δβ3α3-D132A and eIGPS-Δβ7α7-N231A) are shown in Figure 2A and 2B, and the near-UV CD spectra are shown in Figure 2C and 2D. Relatively small changes in the far-UV and near-UV CD spectra are observed for sIGPS-Δβ3α3-D128A, eIGPS-Δβ1α1-S82A and eIGPS-Δβ3α3-D132A compared to their respective WT sequences. However, the significant changes in the near-UV CD spectra for the sIGPS-Δβ2α2-E74A, sIGPS-Δβ7α7-N228A and eIGPS-Δβ7α7-N231A variants imply that the deletion of the β7α7 clamps in both proteins and the β2α2 clamp in sIGPS result in altered aromatic side chain packing.

#### Perturbation of stability by clamp deletion in sIGPS and eIGPS

**Figure 3:**
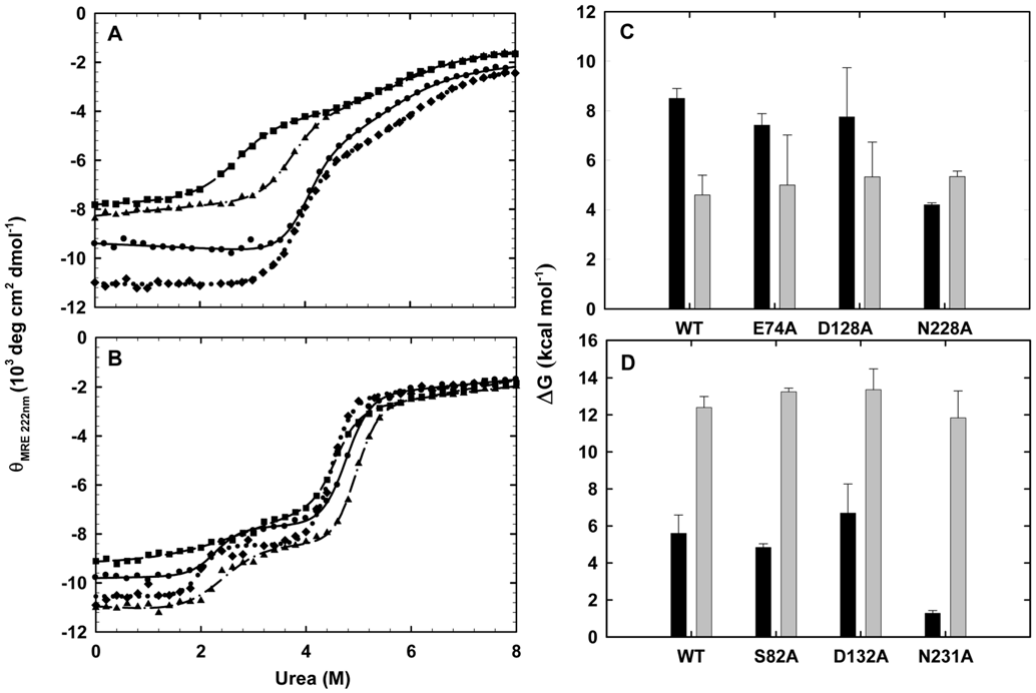
Stability perturbation of sIGPS and eIGPS by clamp deletion. (A) and (B) display urea denaturation equilibrium unfolding curves of WT and clamp-deletion variants of IGPS, the lines represent fits of the data for each variant to a 3-state equilibrium folding model as described in the text. (a) sIGPS: sIGPS-WT (•,–––––), sIGPS-Δβ2α2-E74A (▴,– • –), sIGPS-Δβ3α3-D128A (⧫,•••), and sIGPS-Δβ7α7-N228A (▪,– –). (b) eIGPS: eIGPS-WT (•,–––––), eIGPS-Δβ1α1-S82A (▴,– • –), eIGPS-Δβ3α3-D132A (⧫,•••), and eIGPS-Δβ7α7-N231A (▪,– –). (C) and (D) are bar graphs representing the free energy differences for the N to I step in unfolding, ΔG°_NI_, (black bars) and the I to U step, ΔG°_IU_ (gray bars) for WT and the clamp-deletion variants of sIGPS (C) and eIGPS (D). The urea denaturation equilibrium unfolding curve of sIGPS-WT (A) and the corresponding folding free energy changes (C) are adapted from Forsyth *et al.* [17].

The effect of βα-hairpin clamp deletion on the stability of sIGPS and eIGPS was determined by urea denaturation. As for αTS [16], both sIGPS [17] and eIGPS [18] unfold via a highly populated intermediate, and their unfolding titration curves are well described by a three-state model, N▒ I▒ U. With the exception of eIGPS-Δβ7α7-N231A, the urea-induced unfolding transition for each of the remaining five clamp-deletion variants is also well-described by this three-state model (Figure 3A and 3B). Because a distinct transition between the native state (N) and the intermediate state (I) is not observed during the urea induced denaturation of eIGPS-Δβ7α7-N231A (Figure 3B), kinetic unfolding experiments were performed to verify the existence of I and measure the free energy difference between N and I [6].

The presence of I in eIGPS-Δβ7α7-N231A is verified by the observation of a slow kinetic unfolding phase, whose relaxation times decrease with increasing final denaturant concentration [19], when eIGPS is subjected to an unfolding jump from 0 to 3 M urea where I is expected to be highly populated. Because the amplitude of the unfolding phase is proportional to the population of N from which the reaction initiates, the decrease in the amplitude as a function of increasing initial urea concentrations (Figure S1) can be fit to a two-state model, N ▒ I, to extract the stability, ΔG°_NI_, and the urea dependence of the stability, *m*_NI_ (Dataset S1). These parameters are used to fit the CD unfolding transition for eIGPS-Δβ7α7-N231A (Figure 3B) and to extract ΔG°_IU_ and *m*_IU_ (Methods S1).

The stabilities of N and I for the clamp-deletion variants and the WT parent sequences are illustrated graphically in Figure 3C and 3D for sIGPS and eIGPS, respectively. The free energy differences between N and I, ΔG°_NI_, and between I and the unfolded state, U, ΔG°_IU_, as well as the *m*-values, are tabulated in Table 1. The deletion of the β2α2 clamp in sIGPS, sIGPS-Δβ2α2-E74A, only reduces the stability of N by 1.08 kcal mol^−1^, and the deletion of the β3α3 clamp, sIGPS-Δβ3α3-D128A, has no significant effect on its stability. By striking contrast, the elimination of the β7α7 clamp, sIGPS-Δβ7α7-N228A, reduces the stability of N by 4.30 kcal mol^−1^. Consistent with the absence of these clamps in I for all of these variants, the free energy differences between I and U for the clamp-deletion variants are comparable to the corresponding value for sIGPS-WT (Figure 3C and Table 1). Similar results are obtained for eIGPS. Only eIGPS-Δβ7α7-N231A decreases the stability of N significantly, ΔΔG° = 4.32 kcal mol^−1^. eIGPS-Δβ1α1-S82A and eIGPS-Δβ3α3-D132A have no significant effect on the stability of N, and none of the clamp-deletion variants perturb the stability of I relative to U (Figure 3D and Table 1). Thus, while the elimination of either the β1α1, β2α2 or β3α3 clamps has only marginal effects on sIGPS and eIGPS, the β7α7 clamps in both proteins contribute significantly to both the structure and the stability of the native states for their resident TIM barrel protein.

**Table 1:** Thermodynamic parameters for the urea-induced unfolding of sIGPS, eIGPS, αTS and eight βα-hairpin clamp-deletion variants^a^.

	Donor and acceptor pairs	Donor and acceptor distance (Å)	variants	ΔG°_NI_ (H_2_O) (kcal mol^−1^)	*−m*_NI_ (kcal mol^−1^ M^−1^)	ΔG°_IU_(H_2_O) (kcal mol^−1^)	*−m*_IU_ (kcal mol^−1^ M^−1^)	ΔΔG°_NI_ (kcal mol^−1^)^b^
sIGPS			WT^c^	8.50±0.40	2.10±0.10	4.60±0.80	0.86±0.13	–
	S104 –E74	3.3	E74A	7.42±0.46	1.97±0.13	5.00±2.02	0.86±0.36	−1.08±0.61
	I107-D128	3.0	D128A	7.75±1.99	1.99±0.12	5.33±1.40	0.89±0.23	−0.75±2.03
	K207-N228	2.7	N228A	4.20±0.08	1.56±0.03	5.34±0.22	0.97±0.04	−4.30±0.41
eIGPS			WT	5.60±0.99	2.46±0.42	12.39±0.60	2.60±0.13	–
	F50-S82	3.0	S82A	4.84±0.19	2.05±0.08	13.24±0.20	2.68±0.04	−0.76±1.01
	I111-D132	2.9	D132A	6.69±1.57	3.34±0.77	13.36±1.12	2.99±0.26	1.09±1.86
	V211-N231	2.8	N231A	1.28±0.15^d^	0.89±0.11^c^	11.84±1.45^e^	2.62±0.31^e^	−4.32±1.00
αTS^f^			WT	7.19±0.58	2.85±0.24	3.04±0.85	0.81±0.17	–
	F19 –D46	2.8	D46A	1.98±0.45	0.78±0.17	4.97±1.96	1.07±0.39	−5.21±0.73
	I97-D124	2.6	D124A	2.53±0.40	1.12±0.19	3.81±0.64	0.79±0.16	−4.66±0.70

a Buffer conditions: 10 mM potassium phosphate, 0.2 mM K_2_EDTA, 1 mM βME, pH 7.8 for sIPGS and pH 7.0 for eIGPS at 25°C.

b Perturbation in stability for the N to I reaction, calculated by ΔΔG°_NI_  =  ΔG°_NI_ (H_2_O, variant) − ΔG°_NI_ (H_2_O, WT).

c Values are from Forsyth *et al.* [17].

d Determined by fitting the urea dependence of the amplitude of the unfolding kinetic phase to a two-state model.

e Determined by fitting the equilibrium unfolding data to a three-state model with parameters for the N to I transition fixed to the values determined as described in footnote c.

f Values are from Yang *et al*. [6]

### Survey of βα-hairpin clamps in the TIM barrel proteins

The observation that βα-hairpin clamps can have a significant effect on structure and stability in three TIM barrel proteins motivated a survey of the prevalence of such non-local MC-SC H-bonds in the TIM barrel fold. This analysis was carried out for a structural database of 71 TIM barrel domains, previously reported as a non-redundant representation of the TIM barrel fold [15]. H-bonds between main chain amide hydrogens and polar side chains (MC*_NH_* → SC) that serve as βα-hairpin clamps in the TIM barrel domains were identified (Materials and Methods) for a direct comparison with experimental results.

In the 71 TIM barrel proteins examined, there are 131 MC*_NH_* → SC βα-hairpin clamps. As can be seen in Table S1, there is a very significant preference, >42% of the clamps (χ^2^_Yates_ = 592.49, n = 131, d = 3, p-value = 4.26×10^−128^), for aspartic acid SCs forming H-bonds with the MC amide hydrogen of isoleucine, leucine and valine residues. Inspection of the location of the donor and acceptor residues in the β-strands reveals that every βα-hairpin clamp secures the N-terminus of one β-strand to the loop preceding the subsequent β-strand in the barrel.

**Figure 4:**
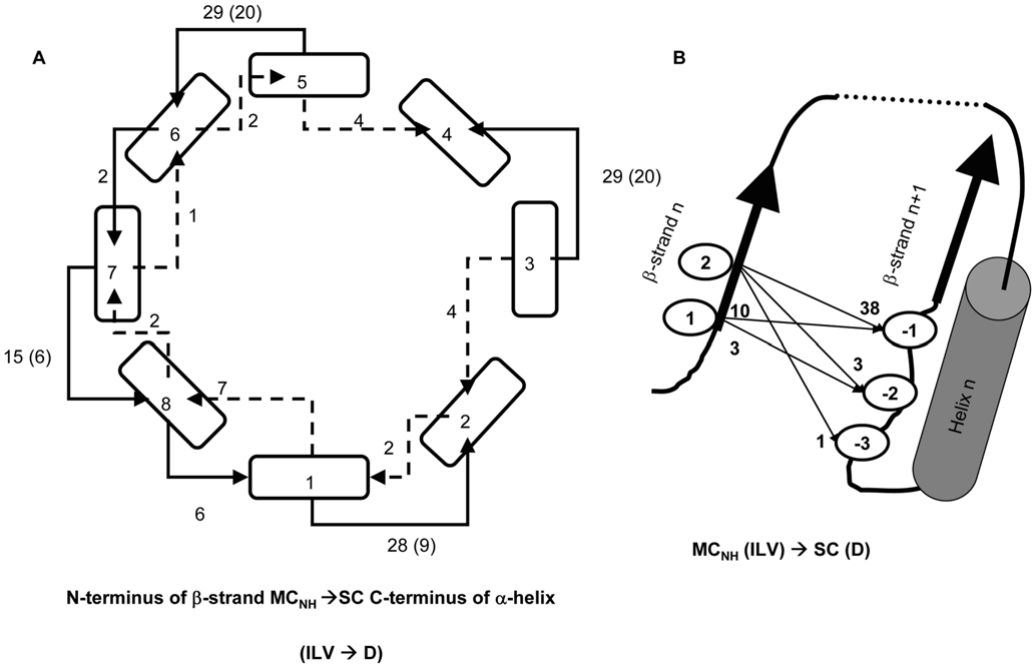
Positional preference of βα-hairpin clamps in 71 TIM barrel proteins. (A) The TIM barrel architecture is represented by a cross-sectional view of the 8 β-strands, represented as rectangles and the strand number is indicated. The number of MC_NH_ → SC βα-hairpin clamp interactions connecting adjacent β-strands with SC H-bond acceptors C-terminal to the MC_NH_ donors (–––––), and with SC H-bond acceptors N-terminal to the MC_NH_ donors (– –) are indicated. The number of βα-hairpin clamps with (I/L/V) MC → SC (D) is represented in parenthesis. (B) The positional preference of (I/L/V) MC → SC (D) relative to the β-strands. The MC donor prefers either the first or second position of the β-strand and the SC acceptor prefers to be in the loop immediately preceding the subsequent β-strand. The number of times each pair of interactions occurs in the 55 I/L/V MC → SC D sub-set is indicated.

The locations of the entire group of 131 MC*_NH_* → SC βα-hairpin clamps are displayed in Figure 4A, with each clamp interaction represented as a bridge across two adjacent β-strands. A very strong preference (77%) is seen for β1α1, β3α3, β5α5 and β7α7 clamps, where the SC acceptor is C-terminal to the MC H-bond donor. With the exception of 13 β8α8 clamps, the paucity of β2α2, β4α4 and β6α6 clamps is distinct from their odd β-strand counterparts. The relatively large number of clamps for the β8β1 interface may reflect the necessity for securing the N- and C-terminal β-strands. Far fewer βα-hairpin clamps, in which the SC acceptor is N-terminal to the MC H-bond donor, are observed. Highlighting the significance of this distribution pattern, the 55 Ile, Leu and Val (I/L/V) MC → SC Asp (D) sub-group of βα-hairpin clamps always have their MC H-bond donor I/L/V located in the odd-numbered stands, β1, β3, β5 or β7, and their SC acceptor, D, is always located before the succeeding even-numbered β-strands, β2, β4, β6 and β8. There is also a strong preference for the I/L/V residue to occupy the 2^nd^ position in the odd-numbered β-strand and for the D residue to occupy the position immediately preceding the even-numbered β-strand (Figure 4B). This positional preference braces two consecutive and adjacent β-strands, along with the intervening helix, and reinforces the β-strand register required for the canonical TIM barrel architecture [20].

## Discussion

Experimental analysis of βα-hairpin clamps between MC H-bond donors and SC H-bond acceptors in three TIM barrel proteins, αTS [6], sIGPS and eIGPS, has shown that a subset of these non-covalent interactions make substantive contributions to stability. Comparisons of the potency of the βα-hairpin clamps in these three proteins shows no correlation between the contributions of these clamps to stability and either the location of the clamps in the structure, their contributing residues or their relative exposure (0–25%) to the solvent. The observation of potent clamps formed by the neutral N228 in sIGPS and N231 in eIGPS, the β7α7 clamps, also shows that the formal negative charge on the aspartic acid H-bond acceptors in the remaining two potent βα-hairpin clamps is not determinative of the strength of the clamp interaction. An examination of the crystal structures of the three proteins, however, suggests that the length of the H-bond in each structure differentiates between the clamps that make major or minor contributions to stability (Table 1). Although the nominal resolutions of the crystal structures of these proteins, 2.0 to 2.8 Å [21]–[23], dictate that the correlation between H-bond length and the clamp contribution to protein stability be viewed as tentative, it appears βα-hairpin clamps whose H-bonds are less than 2.8 Å in length are those, which when replaced with alanine, reduce the stability of the native state by 4-6 kcal mol^−1^. The apparent correlation provides a logical and testable hypothesis for future experiments on βα-hairpin clamps in other TIM barrel proteins.

The assay for the contribution of the βα-hairpin clamps to the stability of three TIM barrel proteins involves the replacement of the polar side chain H-bond acceptors, asparagine, aspartic acid, glutamic acid and serine, with alanine. The absence of the H-bond acceptor moiety is accompanied by the introduction of a potential void for these buried side chains, reflecting the absence of chemical mass as the side chain is truncated to the β-carbon. The perturbations in the secondary and/or tertiary structures induced by the mutations (Figure 2C and 2D) show that the loss of the clamp is propagated to numerous other non-covalent interactions via the global cooperativity of the native conformation.

The absence of the βα-hairpin clamps in the I states of all three TIM barrel proteins demonstrates that the potent effects of these clamps only appear as the N state appears [6]. Kinetic folding studies on αTS revealed further that each clamp is crucial for accessing the transition state ensemble required to reach the properly-folded structure [6]. Although the local connectivity of the βαβ modules might have been expected to enable the clamp to have a role in the early stages of the folding reaction, the primary role of the potent set of clamps is to drive the final stage of the reaction to completion and fully develop global cooperativity.

**Figure 5:**
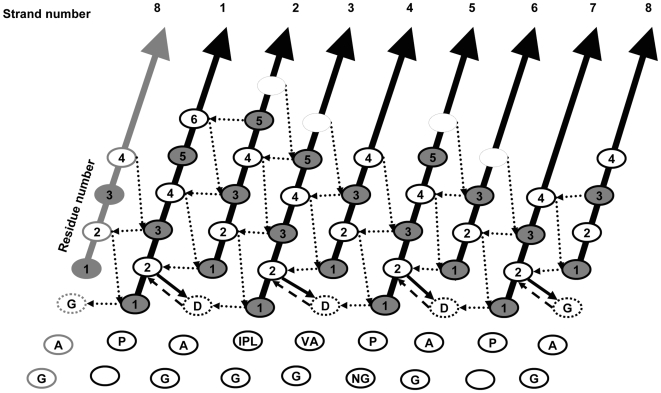
Architectural principles of the TIM barrel fold. The strand number of the 8 β-strands of the TIM barrel architecture (↗) is indicated at the C-terminus of each β-strand. To convey the closed barrel architecture, β8 is shown adjacent to β1 (↗) as well as adjacent to β7. The position of each residue on the β-strands, with SC pointing into the β-barrel (••) and SC pointing towards the α-helices (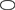), is indicated. The one letter code for the most common amino acids (>15%) in the loop preceding the β-strand in the 71 TIM barrel proteins database is shown. The H-bond network for the β-barrel (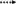), the βα-hairpin clamp interactions between the second residue of an odd-numbered β-strand and the side chain of the residue immediately preceding the subsequent even-numbered β-strand (→), and the MC-MC interactions between the same two residues (− →) are indicated.

The 4-fold symmetry of the preferred βα-hairpin clamps is mirrored, not only in the symmetry of the βαβ modules, but also in the packing of the side chains in the interior of the β-barrel. A residue oriented towards the inside of the β-barrel from an odd-numbered β-strand is at the same level as corresponding residues from the three remaining odd-numbered β-strands. The next layer is comprised of the four side chains from the even-numbered β-strands (Figure 5); the third, and usually final layer, is comprised again of side chains from the odd-numbered β-strands [20]. The layering of side chains inside the barrel has its origin in the tilt of the β-strands (35°) with respect to the central axis of the β-barrel [24]. The resulting S = 8 shear [20], [24] provides a favorable orientation for the H-bonding network between adjacent parallel β-strands and provides opportunities for MC-SC βα-hairpin clamp interactions. Together, these non-covalent interactions and others stabilize the (βα)_8_, TIM barrel fold (Figure 5). The observation of similarly placed non-local MC–SC interactions in a limited survey of flavodoxin fold proteins (data not shown) suggests that βα-hairpin clamps are a common structural feature of βα-repeat proteins.

The chemical origin for the asymmetry between odd- and even-numbered β-strands is apparent from an inspection of the residue preference (>15%) at positions preceding the N-terminus of each β-strand (Figure 5). The conserved proline just before odd-numbered β-strands provides a kink in the backbone that marks the beginning of a β-strand [5]. The preferred sequence pattern of the tight turn connecting the α-helix and the subsequent even-numbered β-strand (Figure 5), GAD, has been reported previously [1]. The positive ϕ angle allowed by glycine and the hydrophobic nature of alanine immediately following the α-helix enables a Schellman motif for the C-terminal capping of the helix [10] and a tight turn to the next β-strand. The aspartic acid just prior to the beginning of even-numbered β-strands forms the βα-hairpin clamp and braces the βαβ module. This N-terminal cap for the odd-numbered β-strand is very often complemented by a MC–MC H-bond, with the amide group of the aspartic acid acting as the donor to the MC carbonyl oxygen of the partner residue. While other SC acceptors are observed (Table S1), the length of the aspartic acid side chain appears to be optimal for the reinforcement of the MC–SC H-bond with the MC–MC H-bond, providing a plausible explanation for its higher frequency in βα-hairpin clamps.

The preference for I/L/V residues at the MC*_NH_* H-bond donor position may reflect, in part, the >40% occurrence of these residues in parallel β-strands of TIM barrel proteins [20]. Further, along with alanine and glycine, I/L/V are the only amino acids that do not partition favorably from the vapor phase to water [25]. As such, these large aliphatic side chains are especially effective at excluding water from MC–SC H-bonds in the βα-hairpin clamps. The exclusion of water, that is apparent from the limited access to solvent for the H-bond donor and acceptor atoms of potent clamp interactions in αTS, sIGPS and eIGPS (Figure 1C and 1D), is expected to strengthen these H-bonds and make them more resistant to exchange with solvent, as observed previously for αTS [26]–[28]. This presumption is supported by the conclusions of Gao *et al.* [29], who recently reported that the strength of a MC–MC H-bond is inversely related to the polarity of its local environment. Valine more effectively screened an underlying β-sheet MC–MC H-bond from solvent than alanine in a Pin WW domain, increasing the strength of the H-bond by up to 1.2 kcal mol^−1^.

The occurrence of the βαβ motif in a large number of protein families [3], [20] suggests that the N-terminal capping of β-strands by βα-hairpin clamps, akin to the analogous N-capping of α-helices [9], [10], may be a useful property for the refinement of protein fold prediction and for engineering stability in βα-repeat proteins. βα-repeat proteins are readily recognized from their sequences and the predicted alternating patterns of α-helices and β-strands [30]. The refinement of the 3D structures predicted from knowledge-based potentials [31], threading [32] and homology modeling [33] of these protein sequences, could be enhanced by screening for βα-hairpin clamps between the MC amide hydrogens at favored positions near the N-terminus of a β-strand and H-bond acceptor SC in the loop before the subsequent β-strand (∼25 residues apart in sequence). These clamps would establish the register of the pair of β-strands, and, with the very short loop linking the intervening α-helix to the second β-strand, it might be possible to establish the register of the α-helix on the β-strand pair in the βαβ module. Although TIM barrel proteins typically contain only a few βα-hairpin clamps, defining the spatial relationships of the components of a subset of βαβ modules might increase the probability of predicting the packing of adjacent βα-repeats in the structures. The effect of accurately predicting the structure of one βαβ module might, therefore, propagate throughout the TIM barrel protein.

The TIM barrel architecture provides a scaffold that is capable of a very diverse set of enzymatic functions [1], and this property has enabled TIM barrel enzymes to be re-engineered in order to accommodate alternative substrates [34]–[37] and even to catalyze non-biological reactions [38]. Because the active sites of TIM barrel enzymes are invariably comprised of the loops protruding from the C-termini of the β-strands, engineering βα-hairpin clamps at the N-termini of the β-strands offer a unique opportunity to enhance the stability of TIM barrel proteins without compromising function.

## Materials and Methods

### Clamp-deletion variants

The plasmid encoding a truncated version of sIGPS, in which the non-canonical additional α-helix (α00) at the N-terminus was deleted to eliminate aggregation during folding, pTNI4 [17], was obtained from Dr. K. Kirschner (University of Basel, Switzerland). The plasmid coding for eIGPS, pJB122 [39], was obtained from Dr. J. M. Blackburn (University of the Western Cape, South Africa). The eIGPS, with an additional Ala residue after the start codon and a C-terminal FLAG peptide sequence (GSDYKDDDDK), is fully folded and catalytically active [39]. Oligonucleotides for mutagenesis were purchased from Eurofins MWG Operon (Huntsville, AL), and the Quickchange^TM^ site-directed mutagenesis kit was obtained from Stratagene (La Jolla, CA). The site-directed mutations were confirmed by DNA sequence analysis (Genewiz Inc, NJ).

### Protein expression and purification

The sIGPS protein and its variants were expressed in BL21/DE3 cells and purified as described previously [17]. The expression and purification of eIGPS and its variants followed the same protocol, with the exception that the procedures were conducted at pH 7.0. The purity (>95%) was demonstrated by the appearance of a single band Coomassie blue stained PAGE and confirmed using electrospray mass spectrometry at the Proteomics Facility at the University of Massachusetts Medical School (Worcester, MA).

### Circular dichroism

Far- and near-UV CD spectroscopy was employed to monitor the secondary and the tertiary structure near aromatic side chains, respectively. Spectra were obtained on a Jasco Model J-810 spectropolarimeter equipped with a thermoelectric cell holder. Far-UV CD data were collected from 280 nm to 185 nm at a scan rate of 50 nm/min and at 1 nm intervals using a 0.1 cm pathlength cell, a bandwidth of 2.5 nm, with an averaging time of 8 s. Three replicate spectra were collected and averaged. The protein concentration was 5 µM. Near-UV CD data were collected from 350 nm to 250 nm at 5 nm/min using a 0.5 cm path length cell, and the protein concentration was 50–150 µM. The temperature was maintained at 25°C with a computer-controlled Peltier system.

### Thermodynamic measurements

The stability of the IGPS clamp-deletion variants was measured by urea denaturation as described previously [17] in a buffer containing 10 mM potassium phosphate, pH 7.8 for sIGPS and pH 7.0 for eIGPS, 0.2 mM K2EDTA, and 1 mM βME. A Hamilton 540B automatic titrator was used to prepare the samples containing 0 to 8 M urea at concentration increments of 0.2 M urea to enhance the precision of the measurements. The samples were incubated overnight at 25°C to ensure equilibration.

### Data analysis

Equilibrium CD data at 222 nm were fit to a three-state model, N ▒ I ▒ U, as described previously [40]. All thermodynamic folding data were fit using Savuka version 6.2, an in-house, non-linear, least-squares program [40].

### Survey of TIM barrel proteins

A database of 71 TIM barrel proteins has been previously developed (http://www.cbrc.jp/~gromiha/tim/proteinlist.html [15]) from the SCOP [41] and HOMSTRAD [42] databases, with a pair-wise sequence homology of <25%. The highest resolution structure for each domain was chosen from the Protein Data Bank [43]. The secondary structure was calculated using the DSSP program [44] and the H-bond interaction parameters were calculated using default settings of the HBPLUS program [45].

### Definitions of βα-hairpin clamp interactions

For each protein, the 8 canonical β-strands and α-helices in the context of the TIM barrel architecture were identified and labeled accordingly. H-bonding partners identified using the HBPLUS program [45], were subjected to the following filters: 1) the H-bonds must be between a MC amide donor and a SC acceptor, 2) the amino acid chain length between the donor and acceptor must be ≥15 residues thereby eliminating shorter-range helix-capping interactions [9], [10] and 3) the chain must include exactly one β-strand and one α-helix identified in the context of the TIM barrel architecture. For the case of the β8α8-hairpin clamps, MC*_NH_* → SC H-bonds between the residues prior to β1 and β8 were included. The H-bonds that passed each stage of the filtering process were exported to a PyM0L [46] script in color-coded fashion for manual confirmation.

### Statistical significance of residue preference for βα-hairpin clamps

The frequency of MC*_NH_* → SC H-bonds in the 71 TIM barrel proteins, where the donor and acceptor residues were at least 15 amino acids apart and were not involved in βα-hairpin clamp interactions, was determined. This frequency was used to calculate the expected frequency of H-bonding between any two types of residues and compared to that observed in βα-hairpin clamps. Four categories, Ile MC → SC D, Leu MC → SC D, Val MC → SC D, Other MC → SC Other, were used to determine the χ^2^ distribution probabilities, with Yates correction [47], of observed βα-hairpin clamps.

## Supporting Information

Dataset S1 Thermodynamic analysis of the eIGPS Δβ7α7 N231A clamp-deletion variant.
(0.04 MB DOC)

Methods S1 Kinetic experiments.
(0.04 MB DOC)

Figure S1 Unfolding amplitude of eIGPS Δ β7α7 N231A as a function of initial urea concentration.
(0.26 MB DOC)

Table S1 Sequence preferences for βα-hairpin clamps in 71 TIM barrel proteins.
(0.07 MB DOC)
