# Supplementary material for: βα-Hairpin Clamps Brace βαβ Modules and Can Make Substantive Contributions to the Stability of TIM Barrel Proteins
Source: PLoS One. 2009 Sep 29;4(9):e7179. doi: 10.1371/journal.pone.0007179 (PMC2747017; doi:10.1371/journal.pone.0007179)
Supplement: Dataset S1 — Thermodynamic analysis of the eIGPS Δβ7α7 N231A clamp-deletion variant. (0.04 MB DOC) [file pone.0007179.s001.doc]

**SUPPLEMENTARY DATA**

*Thermodynamic analysis of the eIGPS‑Δβ7α7‑N231A clamp-deletion variant.*

The presence of the intermediate state, I, for the clamp-deletion variant eIGPS‑Δβ7α7‑N231A was verified, and the free energy difference between its N and I states was measured with a kinetic unfolding experiment. The basis for this experiment relies on the fact that the amplitude of the unfolding reaction is directly proportional to the population of the folded state from which the reaction is initiated. Thus, the stability of the initial folded state can be measured by monitoring the amplitude of the unfolding reaction (to a constant urea concentration) as a function of the initial urea concentration. The amplitude of the unfolding phase is expected to decrease in a sigmoidal fashion as the initial urea concentration is increased into the zone where the native state becomes depopulated. This procedure was validated previously on αTS[6].

A fit of the sigmoidal decrease in ellipticity at increasing initial urea concentrations to a two-state model for eIGPS-WT yielded a stability of 5.29 ± 1.71 kcal mol^-1^ (**Supplementary Fig. 1a**). This value is in excellent agreement with that for the N to I transition from the standard equilibrium titration experiment on the WT protein, 5.60±0.99 kcal mol^-1^ (Figure 3 and Table 1 in the main text). The urea dependence of the stability, the *m*-value, is also in excellent agreement, 2.34 ± 0.74 *vs.* 2.46 ± 0.42 kcal mol^‑1^ M^-1^ (Table 1 in the main text).

Applying this method to the clamp-deletion variant, eIGPS‑Δβ7α7‑N231A (**Supplementary Fig. 1b**), the free energy difference for the N to I transition is 1.28 ± 0.15 kcal mol^-1^ and the *m*-value is 0.89 ± 0.11 kcal mol^-1^ M^-1^. The free energy difference for the I to U transition for eIGPS‑Δβ7α7‑N231A can be estimated by fitting the equilibrium unfolding titration data (Fig. 3c in main text) to a three-state model, N ⮀ I ⮀ U. In this fit, the parameters for the N to I reaction were fixed to those obtained from the kinetic unfolding experiments, and the parameters for the I to U reaction were varied to optimize the fit to the equilibrium unfolding data. The thermodynamic parameters obtained for the I to U transition are shown in Table 1 in the main text.
