# Supplementary material for: βα-Hairpin Clamps Brace βαβ Modules and Can Make Substantive Contributions to the Stability of TIM Barrel Proteins
Source: PLoS One. 2009 Sep 29;4(9):e7179. doi: 10.1371/journal.pone.0007179 (PMC2747017; doi:10.1371/journal.pone.0007179)
Supplement: Methods S1 — Kinetic experiments. (0.04 MB DOC) [file pone.0007179.s002.doc]

**SUPPLEMENTARY METHODS**

*Kinetic experiments.* CD manual-mixing kinetic experiments were performed on a Jasco Model J-810 spectropolarimeter equipped with a thermoelectric cell holder using a 1 cm pathlength cell, a bandwidth of 2.5 nm, and an averaging time of 1 s. The dead-time of the experiments was 3 s, and the instrument response time was about 5 s. The change in ellipticity as a function of time was monitored at 222 nm. Kinetic unfolding experiments to determine the stability of the N state of the clamp-deletion variant were performed by jumping from different initial urea concentration (0-2.8 M) to a final concentration 3 M urea. Protein samples were first equilibrated in the initial urea concentration overnight and then jumped to 3 M urea in buffered solutions by a 1:10 dilution. The final protein concentration was 5 μM.
