# Supplementary material for: βα-Hairpin Clamps Brace βαβ Modules and Can Make Substantive Contributions to the Stability of TIM Barrel Proteins
Source: PLoS One. 2009 Sep 29;4(9):e7179. doi: 10.1371/journal.pone.0007179 (PMC2747017; doi:10.1371/journal.pone.0007179)
Supplement: Figure S1 — Unfolding amplitude of eIGPS Δ β7α7 N231A as a function of initial urea concentration. (0.26 MB DOC) [file pone.0007179.s003.doc]

(a)

(b)

**Supplementary Figure 1.** The dependence of the amplitude for the unfolding phase for (a) eIGPS-WT (●,**──**) and (b) eIGPS-Δβ7α7-N231A (■,– –) on the initial urea concentration; the final urea concentration in all cases was 3 M urea. The lines represent the fit of the data to a two-state model with ΔG° = 5.29 ± 1.71 kcal mol^-1^ and *m* = 2.34 ± 0.74 kcal mol^‑1^M^‑1^ for eIGPS-WT and ΔG° = 1.28 ± 0.15 kcal mol^-1^ and *m* = 0.89 ± 0.11 kcal mol^-1^ M^-1^ for eIGPS-Δβ7α7-N231A.
