# Supplementary material for: βα-Hairpin Clamps Brace βαβ Modules and Can Make Substantive Contributions to the Stability of TIM Barrel Proteins
Source: PLoS One. 2009 Sep 29;4(9):e7179. doi: 10.1371/journal.pone.0007179 (PMC2747017; doi:10.1371/journal.pone.0007179)
Supplement: Table S1 — Sequence preferences for βα-hairpin clamps in 71 TIM barrel proteins. (0.07 MB DOC) [file pone.0007179.s004.doc]

**Supplementary Table 1.** Sequence preferences for MC_NH_🡪SC βα-hairpin clamps in 71 TIM barrel proteins.

**MC_NH_ →**

| **SC↓** | **ALA** | **ARG** | **ASN** | **ASP** | **CYS** | **GLN** | **GLU** | **GLY** | **HIS** | **ILE** | **LEU** | **LYS** | **MET** | **PHE** | **SER** | **THR** | **TRP** | **TYR** | **VAL** |
| --- | --- | --- | --- | --- | --- | --- | --- | --- | --- | --- | --- | --- | --- | --- | --- | --- | --- | --- | --- |
| **ASN** | 1 (1) | 1 (1) | (1) | 1 (1) | 1 (0) | (1) | (1) | 2 (1) | (0) | 4 (2) | 2 (1) | 2 (1) | (0) | (1) | (1) | (1) | (0) | 1 (1) | 2 (1) |
| **ASP** | 2 (2) | 2 (2) | (2) | (2) | 3 (1) | (1) | (1) | (5) | 1 (2) | **18** (1) | **14** (1) | 2 (1) | 2 (1) | 5 (2) | (5) | 2 (3) | (1) | 1 (2) | **23** (3) |
| **GLN** | 1 (1) | (0) | (0) | 1 (1) | (0) | (0) | (0) | (1) | (0) | 1 (1) | 1 (1) | (1) | (0) | 1 (0) | (0) | 2 (1) | (0) | (1) | 1 (1) |
| **GLU** | (3) | 1 (1) | (2) | (2) | (0) | 2 (0) | (1) | (4) | (0) | 2 (1) | 1 (3) | 1 (1) | 1 (1) | 1 (1) | 2 (5) | (2) | (0) | (1) | 1 (1) |
| **HIS** | (0) | (0) | (0) | (0) | (0) | (0) | (0) | (0) | (0) | 1 (0) | 1 (0) | (0) | 1 (0) | (0) | (0) | (0) | (0) | (0) | (0) |
| **SER** | (1) | (1) | (0) | (0) | (0) | (0) | (1) | 2 (3) | (1) | 1 (0) | 2 (1) | (0) | (0) | (1) | (1) | (1) | (0) | (1) | 2 (1) |
| **THR** | (0) | (0) | (1) | 1 (0) | (0) | (0) | (0) | 1 (2) | (0) | 1 (0) | (0) | (0) | (0) | 1 (0) | (1) | 1 (0) | (0) | (0) | 2 (0) |
| **TYR** | (1) | (0) | (0) | (1) | (0) | (1) | (0) | (1) | (0) | (1) | 1 (1) | (0) | (0) | (0) | (1) | (0) | (0) | (0) | (1) |

Numbers in parenthesis are the values expected from the distribution of MC_NH_🡪SC H-bonds in 71 TIM barrel proteins that do not form βα-hairpin clamps and have at least 15 residues between the donor and acceptor residues.
